# The Herd-Level  Sensitivity of Abattoir Surveillance for Bovine Tuberculosis: Simulating the Effects of Current and Potentially Modified Meat Inspection Procedures in Irish Cattle

**DOI:** 10.3389/fvets.2018.00082

**Published:** 2018-05-23

**Authors:** Preben W. Willeberg, Conor G. McAloon, Erik Houtsma, Isabella Higgins, Tracy Ann Clegg, Simon J. More

**Affiliations:** ^1^Department of Diagnostic and Scientific Advice, National Veterinary Institute, Technical University of Denmark, Copenhagen, Denmark; ^2^Section of Herd Health and Animal Husbandry, UCD School of Veterinary Medicine, University College Dublin, Dublin, Ireland; ^3^Centre for Veterinary Epidemiology and Risk Analysis, UCD School of Veterinary Medicine, University College Dublin, Dublin, Ireland

**Keywords:** bovine tuberculosis, simulation modelling, abattoir surveillance, herd sensitivity, Ireland

## Abstract

The European Food Safety Authority (EFSA) has published a series of opinions to assess the impact of changing from the current meat inspection procedures (CMI) to visual-only inspection (VOI) procedures. Concern has been raised that changes from CMI to VOI would adversely affect the effectiveness of surveillance for bovine tuberculosis (bTB) in EU member states, both for countries with and without official status of bTB freedom (OTF and non-OTF countries, respectively). This study was conducted to estimate the impact of a change from CMI to VOI in abattoirs on herd-level detection sensitivity in Ireland, a non-OTF country. Using national Irish data, we identified all herds that sold at least one animal to slaughter during 2010–12 whilst unrestricted for bTB. For each of these herds, we calculated the number of cattle sent to slaughter whilst unrestricted, the number of factory lesion tests (FLT) that had been performed, and estimated the apparent within-herd prevalence (AP_wh_). A FLT is a whole-herd test conducted in a herd following the confirmation of bTB in an animal at slaughter. We considered five different inspection scenarios, each based on meat inspection and bacteriology in series, including current meat inspection (CMI) and four visual-only inspection scenarios (VOI2, VOI3, VOI4, VOI5) with reducing inspection sensitivities. Separately for each inspection scenario, a simulation model was used to estimate the herd-level detection sensitivity and the number of bTB-herds (that is, herds that sent at least one animal detected with *M. bovis* to slaughter when unrestricted during 2010–12) that would and would not be detected. The simulated mean herd-level detection sensitivity estimates were 0.24 for CMI, and 0.16, 0.12, 0.10 and 0.08 for VOI2-5, assuming a 2-, 3-, 4- and 5-fold decrease, respectively, in the animal-level detection sensitivity of VOI relative to that of CMI. The estimated number of non-detected bTB-herds is substantial with CMI, and increases in the series of VOI scenarios with decreasing herd-level detection sensitivity. If VOI were introduced without alternative surveillance means to compensate for the decrease in animal-level inspection sensitivity, such changes might jeopardise bTB surveillance, control and eradication programmes in cattle herds of non-OTF countries, including Ireland.

## 1. Introduction

Meat inspection has the dual purpose of contributing to safeguard public health and food safety as well as to enable surveillance of animal health and welfare (1–4), and both aims should be duly supported by the slaughter-inspection procedures applicable at any time.

In 2012 and 2013, the European Food Safety Authority (EFSA) published a series of opinions considering changes within the European Union from the current meat inspection (CMI) procedures to visual-only inspection procedures (VOI) (1, 5–8). These changes were proposed to increase the safety of products from the main meat-producing species by minimizing the risk of microbiological cross-contamination, e.g., caused by palpation and incision of carcasses (1, 2, 5–8). In these opinions, the potential for negative effects on animal health surveillance were evaluated by EFSA Animal Health and Welfare (AHAW) Panel, and concerns were raised that changes from CMI to VOI might adversely affect the effectiveness of surveillance for bovine tuberculosis (bTB) among the EU member states. This would be particularly problematic in cattle herds, where meat inspection is an important component of bTB surveillance (9, 10), both in countries with and without official tuberculosis freedom (OTF) status.

In OTF countries, surveillance for bTB is undertaken in abattoirs for early detection of recurrent infection and to provide evidence of freedom. With respect to the opinion on inspection of meat from bovine animals, the main focus of EFSA’s AHAW Panel EFSA was on the potential detrimental effects of VOI on substantiating OTF country status (8). The AHAW Panel recommended that the current inspection tasks aimed at detecting bTB should be retained to avoid any reduction in the sensitivity of the overall surveillance system (8). In recent work, Foddai et al. (11) evaluated the impact in Denmark as an OTF country of a potential change in meat inspection from CMI to VOI. They found that a high level of confidence in bTB freedom could be both achieved and maintained if a VOI system were to replace CMI in Danish abattoirs, provided the annual probability of bTB introduction was kept low (e.g., <1%). The apparent discrepancy to the above AHAW Panel evaluation and recommendation was also examined and discussed, and it was ultimately explained by differences in the statistical approaches used to substantiate bTB free status. In addition, it was critical that the bTB-negative Danish results had been accumulated or were assumed across an extended timespan, i.e., a total of 42 years, versus the single-year periods applied in the EFSA model according to the current OTF regulation.

In non-OTF countries, such as Ireland, both field and abattoir bTB surveillance are conducted. In Ireland, all herds are subjected to field surveillance at least once yearly, but more frequently if considered at higher risk. At this test, all animals more than 6 weeks of age are subjected to the single intradermal comparative tuberculin test (SICTT), and positive animals (noting that test interpretation can vary depending on herd risk status) are deemed “reactors”. In addition, all cattle are subjected to post mortem inspection for bTB at an abattoir (so-called abattoir surveillance; an abattoir is also known as a “factory” or slaughterhouse) at the time of slaughter (12). With both field and abattoir surveillance, a bTB “breakdown” (the detection of bTB-infected animals) leads to a period of “herd restriction” with legally binding restrictions placed on cattle in- and out-movements for a period. In non-OTF countries, field surveillance is generally considered the primary method of bTB surveillance. However, abattoir surveillance also plays an important role in national eradication programmes, for two reasons. Firstly, abattoir surveillance can allow early detection of infected herds, prior to the next scheduled skin test (either annual in low risk herds, or more frequent in higher risk herds). This may limit within-herd transmission and between-herd spread, particularly if there is a prolonged period to the next skin test (13). Secondly, abattoir surveillance can facilitate the detection of infection in herds with one or more animals with bTB lesions but without animals positive to the skin test. In Ireland, of the bTB breakdowns triggered by abattoir surveillance, approximately 80% disclose no further reactors during follow-up field surveillance (14). It is likely that many of these animals were infected, but tested false negative at earlier skin tests. Residual infection (the presence of infected, but skin test negative, animals) is an important source of bTB persistence in Irish herds (15). A substantial proportion of bTB breakdowns in both Ireland and the UK were detected during abattoir surveillance: 36% in Ireland in 2006, 15.2% in England in 2006, 14% in Northern Ireland in 2004 (16).

A change from CMI to VOI would lead to a reduction in animal-level inspection sensitivity (the probability that bTB-like lesions will be observed in a bTB-infected animal during abattoir surveillance, see Table 1). The exact magnitude of this reduction cannot be estimated at this point, since potential new alternative VOI procedures and their inspection sensitivity with respect to bTB-like lesions are currently not known. However, a 3- to 5-fold reduction was suggested by EFSA’s AHAW Panel (8), partly based on a scientific report produced by an expert group using a case study approach with France as the example country (17). The detection of bTB during abattoir surveillance relies on the use of several tests in series: meat inspection and, if suspect bTB-like lesions are observed, subsequent laboratory confirmation. For this reason, it is more appropriate to consider animal-level detection sensitivity (the probability that in an infected animal bTB-like lesions will be observed and *Mycobacterium bovis* infection (the infectious cause of bTB) will be detected subsequently through follow-up laboratory testing) (see Table 1). As a contribution to the characteristics of the national bTB surveillance programme, it is also important to estimate the herd-level detection sensitivity (the probability that *M. bovis* will be suspected and confirmed in at least one animal from an infected but non-restricted herd during abattoir surveillance, see Table 1). According to principles outlined previously (18, 19), the herd-level detection sensitivity of bTB depends not only on the animal-level detection sensitivity, but also on the number of cattle from the herd that were sent to slaughter and subject to abattoir surveillance during the given time-period, and of the apparent within-herd prevalence (AP_wh_) of cattle (the percentage of animals slaughtered from the herd that were test-positive, see Table 1).

**Table 1:** Glossary of key terms.

Animal-level inspection sensitivity	The probability that bTB-like lesions would be observed in a bTB-infected animal during abattoir inspection
Animal-level detection sensitivity	The joint probability that bTB-like lesions would be observed in a bTB-infected animal during abattoir inspection and *Mycobacterium bovis* infection would be detected by subsequent laboratory testing
Apparent herd prevalence (AP_h_)	The percentage of herds in which at least one animal would be detected with *M. bovis* at slaughter
Apparent within-herd prevalence (AP_wh_)	The percentage of animals with confirmed *M. bovis* among all animals sent to slaughter whilst unrestricted. AP_wh_ was calculated separately for each eligible herd
bTB	Bovine tuberculosis, caused by infection with *M. bovis*
bTB-herd	A herd unknowingly infected with *M. bovis* that sent at least one animal to slaughter when unrestricted during 2010–12
Eligible herd	All herds that sold at least one animal to slaughter during 2010–12 whilst unrestricted
FLT	Factory Lesion Test, which is the application of the single intradermal comparative tuberculin test (SICTT) on all animals >6 weeks of age on the day of the test, subsequent to a confirmed bTB lesion detected during abattoir surveillance
Herd-level detection sensitivity (HSe)	The probability that *M. bovis* would be detected during abattoir surveillance in at least one animal from a bTB- infected but non-restricted herd
Herd restriction	A period during which the outward movement of cattle from a herd is prohibited, except to slaughter, subsequent to the detection of bTB during field or abattoir surveillance (see also description in Section 2.1)
SICTT	The Single Intradermal Comparative Tuberculin Test
Study herd	All eligible herds, but after excluding herds where the number of animals slaughtered during 2010–12 whilst unrestricted was outside two standard deviations from the mean
True herd prevalence (TP_h_)	The percentage of unrestricted herds during 2010–12 with at least one animal infected with *M. bovis* at slaughter
True within-herd prevalence (TP_wh_)	The percentage of infected animals within eligible herds

This study was conducted to estimate the impact of a presumed change from CMI to VOI in Irish abattoirs on herd-level detection sensitivity during 2010 to 2012.

## 2. Materials and Methods

### 2.1. The Irish Cattle Industry and the National bTB Eradication Programme

At the end of 2012, there were 6.2 million cattle in 107,308 herds in Ireland, with an average herd size of 58 animals (20). Dairying and beef production are important contributors to the national economy, and each is highly dependent on exports. In 2012, exports of Irish dairy products and ingredients were valued at €2.7 billion, and the value of beef exports was almost €3.1 billion (21). Livestock markets are an important component of national trade. In 2012, there were over 2.7 million cattle movements other than directly from farm-to-slaughter, including 60.1% via a market (20).

There has been a national bTB eradication programme in Ireland for many years. All cattle are subjected to both field and abattoir surveillance, as described previously. In recent years, substantial progress is being made. The number of bTB reactors and herd incidence were each lower in 2013 than in any preceding year of the national programme. The role of wildlife is recognised, and progress is being made towards a bTB vaccination for badgers (22, 23). Several authors have highlighted ongoing challenges faced in the programme (15, 24).

### 2.2. Abattoir Surveillance for bTB in Ireland

There are some differences in abattoir surveillance for cattle originating from non-restricted and restricted herds. Cattle from non-restricted herds can be slaughtered at any abattoir (also termed a “factory” or slaughterhouse) in Ireland. When a bTB-like lesion is observed in an animal slaughtered from a non-restricted herd, laboratory confirmation is undertaken, using either histopathology or culture. If bTB is detected, the herd is restricted and a whole-herd “factory lesion test” (FLT, also known as a test type 9a, TT9a) is conducted in the herd from which the lesioned animal had most-recently been resident, this being the application of the SICTT on all animals >6 weeks of age on the day of the test (12). In restricted herds, different procedures are undertaken with skin test positive (“reactor”) and non-reactor cattle. Reactors are slaughtered in designated abattoirs, as defined by the Department of Agriculture, Food and the Marine (DAFM), and no laboratory confirmation is conducted if bTB-like lesions are observed at slaughter. Non-reactor cattle can be slaughtered at any abattoir, with confirmation being conducted following lesion identification.

### 2.3. Estimating Animal-Level Inspection and Detection Sensitivities

We conducted a narrative review of published literature relating to the sensitivity and specificity of meat inspection. We limited our review to studies with a gold standard for sensitivity and specificity estimates of detailed laboratory examination and infection-free cattle populations, respectively, and to studies using Bayesian (no gold standard) methods. Further, apart from one seminal study (25), we focused on recent publications (since 2000). We first considered the results of a systematic review, current to 1 December 2008 (26), in which the final author was involved, and then used a “snowball” method to identify additional references, both in the past (references cited in each of these papers) and the future (references cited by Google Scholar) as at 1 December 2014. These results, with minor modifications, have also been reported elsewhere (27) and subsequently (28, 29). Estimates of the sensitivity and specificity of confirmatory testing of suspect bTB lesions (histology, bacteriology) were as reported in recent publications using Bayesian methods (26–31).

Estimates of the relative sensitivity of meat inspection (when compared with detailed laboratory examination) vary widely, including 9.5% (32), 28.2% (33), 29.4% (34), 36.5% (25) and 55% (35). In comparison to culture and histopathology, Biffa et al. (33) estimated a relative sensitivity of 55.2% for meat inspection. Further recent work has suggested a sensitivity for meat inspection of 67% (measure of central tendency not stated), using a Bayesian partial-likelihood approach (31), a median of 71% using a meta-analysis and latent class analysis (26, 27), and 95% credible intervals of 35.9–42.3% in Ireland, 54.3–63.2% in Northern Ireland and 62.0–87.1% in Spain, using a Bayesian latent class analysis (36). The median of these point estimates is 55%, but with very substantial variation (range of 9.5–87.1%). The specificity of meat inspection is imperfect, given the potential for granuloma-like, but bTB-negative, lesions detectable during abattoir surveillance (37), with specificity estimates of 99.3, 99.6 and 99.9% from Ethiopia (32), Canada (38) and Australia (39), respectively. Using a Bayesian latent class analysis, the estimated median specificity of meat inspection was 97.4–98.5% (95% credible interval) in Ireland, 98.8–99.7% in Northern Ireland and 97.7–98.7% in Spain (36).

The median sensitivity of bacteriology was estimated at 76–79% in the meta-analysis and latent class analysis (31), although this will vary, depending on a range of factors, including aspects of the bacteriological diagnosis chain such as temperature and duration of storage and use of preservatives (40, 41). Although not considered here, very similar estimates were obtained (median 74%, 95% credibility interval 46–94%) in a later analysis where further data were incorporated (28, 29). The assumed specificity of bacteriology is 100%, in the absence of handling and laboratory error. The median sensitivity and specificity of histopathology was estimated to be 63–66% and 100%, respectively (28).

Based on the information above, we calculated an estimated median sensitivity and specificity for meat inspection of 55% (9.5–87.1%) and 99.3% (97.4–99.9%), respectively, and of bacteriology of 77 and 100%, respectively. Assuming independence between these two diagnostic methodologies, the animal-level detection sensitivity of the meat inspection and bacteriology in series for a bTB-affected carcass will be low (42% (that is, 55% × 77%), 7–67%) and the animal-level detection specificity very high (approaching 100%). Very similar results would be expected if we were to consider meat inspection and histopathology in series. Therefore, with a 5-fold drop in animal-level inspection sensitivity, the estimated median animal-level detection sensitivity would be 8% (that is, 0.20 × 55% × 77%).

### 2.4. Estimating Number of Animals Slaughtered and Apparent Within-Herd bTB Prevalence

In Ireland, DAFM manages the Animal Herd Computer System (AHCS), which contains a unique record of each bTB-related testing event in each herd nationally. AHCS integrates with a number of other systems, including the Animal Identification and Movement system (AIM), which stores a computerised record of all movements in and out of all herds nationally, including movement to slaughter (12).

Using methods described previously (42), an episode file was created from the AHCS summary herd-level test data. The episode file represents an aggregation of the raw test data to identify periods of bTB-related herd restriction and non-restriction for all Irish cattle herds during 2010–12. Then, the episode file and AIM database were compared to identify herds that sold at least one animal to slaughter during 2010–12 whilst unrestricted (so-called eligible herds). All other herds were excluded.

For each eligible herd, we interrogated these data using SAS v9.3 (SAS Institute, Cary, NC, USA) to identify:

1. The number of animals sent to slaughter whilst the eligible herd was unrestricted, A. The resulting distribution was explored using SAS proc univariate. The observed number of eligible herds by each of the recorded number of cattle slaughtered during 2010–12 were ranked and listed (from 1 to 24,403 cattle slaughtered, a total of 1,070 unique numbers).
2. Those animals that were detected as bTB-positive based on meat inspection and subsequent laboratory confirmation, have not been directly recorded in the national database. Rather, we recorded the number of FLTs, B, performed for each eligible herd during 2010–12, and for the herds with one or more FLTs we estimated the AP_wh_ as B/A (as defined above), based on the assumption that each FLT was triggered by a single bTB positive slaughtered animal.

Data for herds that slaughtered more or less than 2 SD from the mean were excluded. This was undertaken to eliminate the largest suppliers of slaughter cattle, which are likely to be cattle dealers, feedlots, assembly herds, etc., rather than proper cattle herds. Within the Irish programme, a feedlot herd is defined as “a specialist finisher of beef that does not deliberately engage in the active breeding of animals, notwithstanding that an occasional cow/heifer may calve because it was pregnant on arrival to the feedlot”. In contrast to other herds, there can be fewer restrictions on movement of cattle into restricted feedlot herds depending on the epidemiological circumstances, noting that all animals from these herds are destined for slaughter (12). The number of eligible herds were thus reduced by 248 herds from 86,164 to 85,916 herds (the latter are subsequently termed study herds) (Table 2).

**Table 2:** Descriptive statistics of the Irish data set studied in the analyses of bTB in cattle and cattle herds at slaughter during 2010–12.

Variable	Count	Mean	Median	Mode	Minimum	Maximum
*Eligible*/Study herds*	*86,164*/85,916	n.a.	n.a.	n.a.	n.a.	n.a.
Eligible herds with at least one FLT*^,†^	4,043	n.a.	n.a.	n.a.	n.a.	n.a.
Animals slaughtered from eligible herds	4,338,380	48	12	1	1	24,403
Animals slaughtered from eligible herds with at least one FLT^†^	831,908	206	78	1	1	6,279
AP_wh_, all eligible herds	n.a.	0.0034	0	0	0	1
AP_wh_ of bTB, eligible herds with at least one FLT^†^	n.a.	0.0722	0.0118	0.2000	0.0001	1

*See Section 2.3 for explanation of eligible/study herds.

^†^Factory Lesion Test; see Table 3 for breakdown of data by the number of FLTs.

Unless indicated otherwise, all analyses involving simulations and results thereof have reference to these 85,916 study herds, being herds (except dealers, markets, etc.) that sold at least one animal to slaughter during 2010–12 whilst unrestricted.

For the purpose of estimating herd-level detection sensitivity (HSe), the distribution of true within-herd bTB prevalence within infected herds is required. To achieve this, herds were first filtered to include only those that had at least one FLT. To avoid inflating within-herd prevalence due to small denominators, we dropped those herds that had sent less than 20 animals to slaughter whilst unrestricted. Next, the apparent within-herd prevalence for each infected herd was calculated as nFLT/nSlaughtered. The true within-herd prevalence for each of these herds was then estimated using Rogan-Gladden estimation (43) with animal-level detection sensitivity and specificity of meat inspection and bacteriology in series of 0.42 and 1.0, respectively. Finally, a beta distribution was fitted to the values using the fitdistrplus package in R-studio version 1.0.136 (44).

### 2.5. Estimating Herd-Level Detection Sensitivity

A simulation model was created to estimate HSe (see Appendix 1). For ease of computation, 5,000 herds were randomly sampled from the overall dataset and used for the simulation using the sample function in R. A model with the following structure was then created:

Herd.Inf*_i_* ~ Bernoulli(µ)

$${TP}_{{wh}_{i}}∼Beta(\alpha ,\beta )$$

$${AP}_{{wh}_{i}}∼{Herd.Inf}_{i}\times {TP}_{{wh}_{i}}\times ASe$$

$${nPosSlaughter}_{i}∼Binomial({nSlaughtered}_{i},{AP}_{{wh}_{i}})$$

Where:

- µ is the true herd-level prevalence (TP_h_), i.e., the probability that a randomly selected herd contains ≥1 infected animal. Initially this value was set at 0.052 as the proportion of herds with ≥1 reactor in the dataset. However, since the animal-level detection specificity of abattoir surveillance is assumed to be 1.0 and the probability of a test-positive in the iterations where the herd is uninfected is 0, these iterations have no impact on HSe and will decrease the efficiency of the simulation. Therefore, in order to aid with computation, µ was set to 1.0.
- ${TP}_{{wh}_{i}}$ is the true within-herd prevalence for the* i*-th herd which follows a beta distribution. From the steps described above, a beta distribution with parameters α and β, equal to 1.26 and 37.93 respectively was used for the simulation.
- ${AP}_{{wh}_{i}}$ was the apparent prevalence within the *i*-th herd, which was deemed a product of the binary value Herd.Inf_*i*_, ${TP}_{{wh}_{i}}$ and the animal-level detection sensitivity (ASe) of abattoir surveillance. The animal-level detection specificity of abattoir surveillance was assumed to be 1, so it was not included in the calculation.

For each herd, the number of iterations in which the herd was infected and the number of iterations the herd was detected at slaughter was recorded. HSe was calculated as the proportion of times in which an infected herd was detected at slaughter. The simulation was run for 1,000 iterations over 5,000 sample herds and was implemented in R version 1.0.136 (44).

We considered 5 different inspection scenarios, each based on meat inspection and bacteriology (rather than meat inspection and histopathology) in series: CMI, VOI2 (being a 2-fold decrease in the animal-level inspection sensitivity – and also animal-level detection sensitivity, assuming independence between meat inspection and bacteriology – compared to CMI), VOI3 (3-fold decrease), VOI4 (4-fold decrease) and VOI5 (5-fold decrease). Similarly, given uncertainty over the ASe of abattoir surveillance, the baseline CMI ASe was varied by ±10% and the analysis repeated for each of the 5 scenarios.

### 2.6. Estimating the Number of bTB-Herds Detected During Abattoir Surveillance

From the simulated herd sensitivity values, we estimated the number of bTB-herds (that is, herds infected with *M. bovis* that sent at least one animal to slaughter when unrestricted during 2010–12) that were detected and those that were not detected for each of the inspection scenarios as follows:

The apparent herd prevalence (AP_h_) of bTB was estimated as the proportion of study herds with at least one FLT out of all study herds:

AP_h_ = number of herds with at least one FLT/total number of study herds

Dividing this proportion by the mean herd sensitivity (HSe) estimate for CMI gives an estimate of the true herd prevalence (TP_h_) of bTB:

TP_h_ = AP_h_/HSe(CMI)

Multiplying this with the number of study herds yields the estimated number of bTB-herds:

Number of bTB-herds = total number of study herds × TP_h_

Separately for each inspection scenario, each herd in the overall dataset (*n* = 85,916) was randomly assigned an infection status (1,0) using a Bernoulli distribution with a probability equal to the TP_h_ (above). Each infected herd was then assigned a test (+ve/−ve) status using a Bernoulli distribution with the probability equal to a HSe sampled at random from distribution of simulated HSe values. For each scenario, the number of infected herds and the number of herds that were detected was summed across all herds.

## 3. Results

### 3.1.Descriptive Statistics

Summary statistics on the number of animals sent to slaughter whilst the herds were un-restricted throughout the period 2010–2012 are presented in Table 2. The apparent herd prevalence, AP_h_, was 4.7%. Substantially more animals were slaughtered from herds with at least one FLT during the study period compared with all study herds (mean: 206 vs. 48; median: 78 vs. 12, respectively). It is also noteworthy, that the mode values for the number of cattle slaughtered from herds with at least one FLT and from all herds are both 1. In each case, the herd-size distribution is extremely skewed, since a large proportion of herds provided just a single animal for slaughter during the 3-year-period.

The observed AP_wh_ across all herds for the 3 year period was 0.0034 (Table 2), which is influenced by the large proportion of herds from which only one or very few animals were sent to slaughter. For 95.3% of these unrestricted herds, no bTB-positive animals were detected during abattoir surveillance. The corresponding AP_wh_ for the 4,043 herds with at least one FLT was 0.0722; that is, on average one in fourteen cattle slaughtered from these herds had a confirmed bTB lesion.

Table 3 documents the apparent within-herd prevalence by the number of recorded FLTs, the maximum being four FLTs during the three-year period. Also, descriptive statistics of the four FLT strata as far as the number of herds and their size are presented. For both parameters substantial differences appear between the four FLT strata, with tendencies to higher average number of animals slaughtered and lower average apparent within-herd prevalence as the number of FLTs increase, but the differences are not entirely consistent.

**Table 3:** Number of eligible bTB-herds, AP_wh_ and animals slaughtered by number of factory lesion tests (FLTs) conducted during 2010–2012.

FLTs	Herds		AP_wh_				Animals slaughtered			
	Number	%	Mean	Minimum	Median	Maximum	Mean	Minimum	Median	Maximum
0	82,121	95.30	0.000	0.0000	0.000	0.00	40	1	11	24,403
1	3,679	4.30	0.076	0.0002	0.014	1.00	173	1	69	6,279
2	309	0.40	0.033	0.0007	0.007	0.67	471	3	570	3,065
3	48	0.06	0.021	0.0010	0.004	0.75	948	4	769	3,131
4	7	0.01	0.007	0.0017	0.006	0.01	834	313	623	2,423
1–4	4,043	4.70	0.072	0.0002	0.012	1.00	206	1	78	6,279
All herds	86,164	100	0.003	0.0002	0.000	1.00	48	1	12	24,403

### 3.2. Simulation Results

#### a. Herd-Level Detection Sensitivities

**Figure 1:**
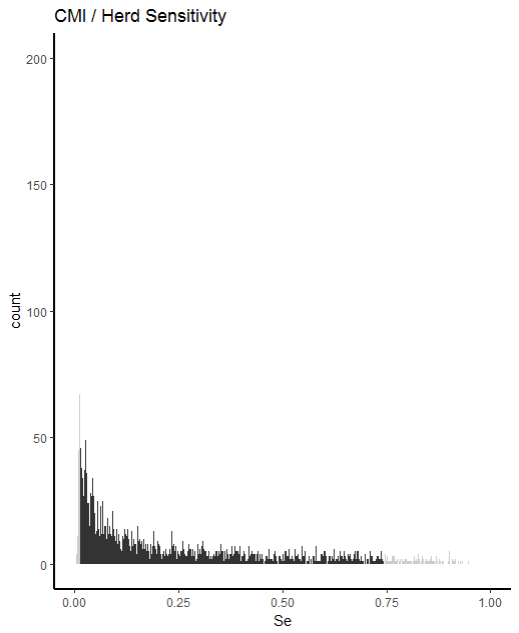
Distribution of herd-level detection sensitivities for CMI (current meat inspection), based on the number of bTB-affected herds among 1,000 simulated Irish herds. The middle 90% of the distribution is highlighted in black, and the 5% tail at either end in grey.

**Figure 2:**
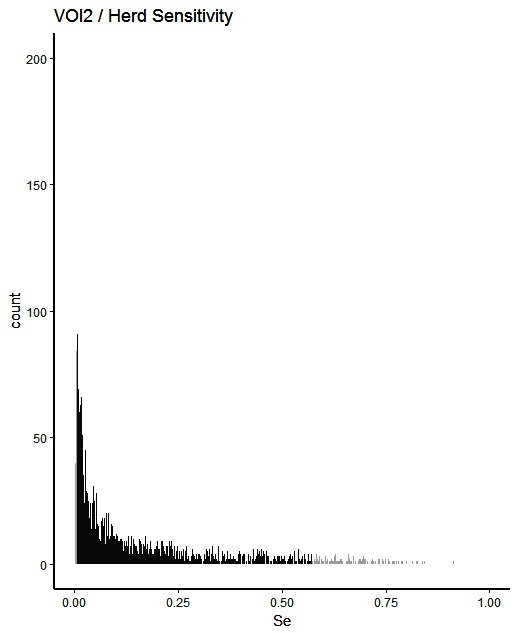
Distribution of herd-level detection sensitivities for VOI2 (visual-only inspection, with a 2-fold reduction in animal-level sensitivities), based on the number of bTB-affected herds among 1,000 simulated Irish herds. The middle 90% of the distribution is highlighted in black, and the 5% tail at either end in grey.

**Figure 3:**
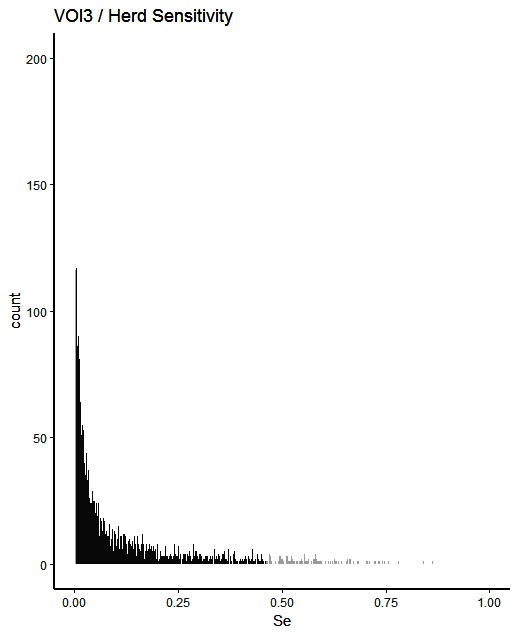
Distribution of herd-level detection sensitivities for VOI3 (visual-only inspection, with a 3-fold reduction in animal-level sensitivities), based on the number of bTB-affected herds among 1,000 simulated Irish herds. The middle 90% of the distribution is highlighted in black, and the 5% tail at either end in grey.

**Figure 4 F4:**
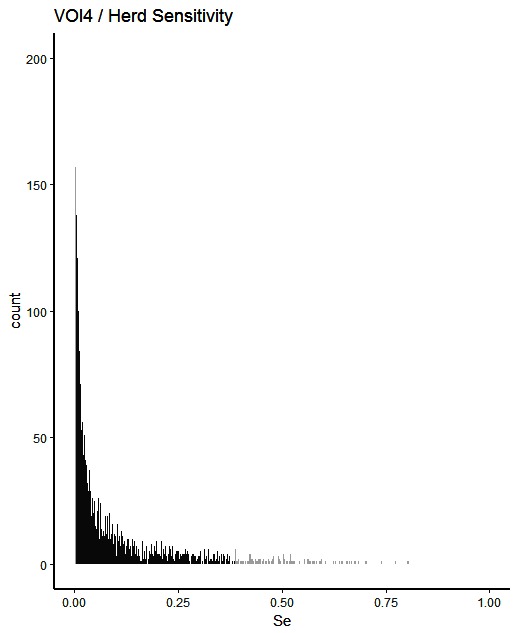
**[Fig F4]**Distribution of herd-level detection sensitivities for VOI4 (visual-only inspection, with a 4-fold reduction in animal-level sensitivities), based on the number of bTB-affected herds among 1,000 simulated Irish herds. The middle 90% of the distribution is highlighted in black, and the 5% tail at either end in grey.

**Figure 5:**
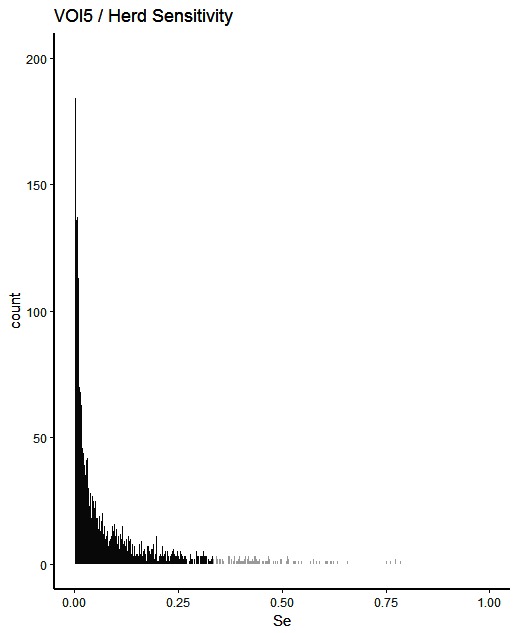
Distribution of herd-level detection sensitivities for VOI5 (visual-only inspection, with a 5-fold reduction in animal-level sensitivities), based on the number of bTB-affected herds among 1,000 simulated Irish herds. The middle 90% of the distribution is highlighted in black, and the 5% tail at either end in grey.

**Figure 6:**
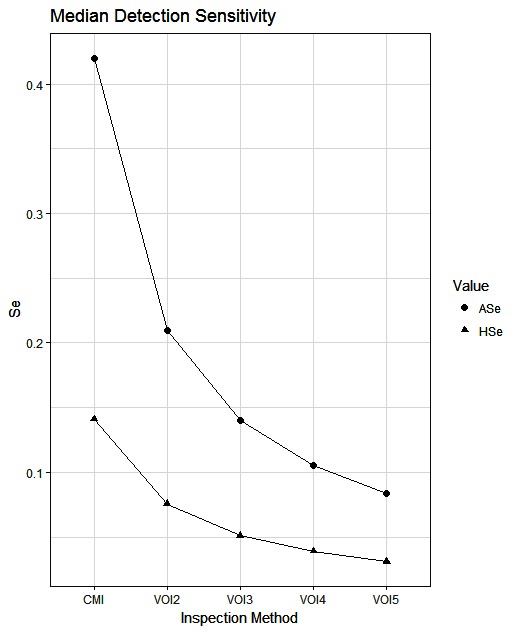
Medians of the simulated distributions for animal- and herd-level detection sensitivity for the five alternative inspection scenarios. Each point is based on the number of bTB-affected herds among 1,000 simulated Irish herds.

The simulated animal- and herd-level detection sensitivities for each of the five inspection scenarios, and the distribution of the herd-level detection sensitivities, are presented in Table 4 and Figures 1–5, respectively. In Table 4, both the mean and the median sensitivities are presented. Figure 6 illustrates the gradual changes in the median animal- and herd-level detection sensitivity by inspection scenario.

**Table 4:** Simulated animal- and herd-level detection sensitivities by inspection scenario, using parameters estimated from bTB slaughter-inspection findings in Ireland during 2010–12.

Baseline animal-level detection sensitivity	Inspection scenario	Detection sensitivity			Estimated number of bTB-herds*		
		Animal-level	Herd-level		Total	Detected	Additional non-detected
			Median	Mean			
0.42	CMI	0.42	0.14	0.24	16,335	3,904	-
	VOI2	0.21	0.08	0.16	16,243	2,540	1,272
	VOI3	0.14	0.05	0.12	16,241	1,930	1,880
	VOI4	0.11	0.04	0.10	16,234	1,580	2,223
	VOI5	0.09	0.03	0.08	16,235	1,349	2,455
0.46	CMI	0.46	0.15	0.25	16,243	4,073	-
	VOI2	0.23	0.08	0.17	16,243	2,692	1,381
	VOI3	0.15	0.06	0.13	16,241	2,080	1,991
	VOI4	0.12	0.04	0.10	16,233	1,687	2,376
	VOI5	0.09	0.03	0.09	16,235	1,439	2,626
0.38	CMI	0.38	0.13	0.23	16,243	3,671	-
	VOI2	0.19	0.07	0.15	16,241	2,396	1,273
	VOI3	0.13	0.05	0.11	16,237	1,788	1,877
	VOI4	0.09	0.04	0.09	16,235	1,461	2,202
	VOI5	0.08	0.03	0.08	16,229	1,239	2,418

*A herd unknowingly infected with M. bovis that sent at least one animal to slaughter when unrestricted during 2010–12.

#### b. Number of Infected Herds Detected and Non-Detected During Abattoir Surveillance

The herd-level detection sensitivity has direct consequences for the number of bTB-herds that would be detected at slaughter and those that would remain undetected. Table 4 presents the estimated numbers of detected and non-detected bTB-herds based on simulations of the 3 × 5 alternative inspection scenarios. The estimated number of non-detected bTB-herds is substantial with CMI, and increases considerably with the decreasing herd-level detection sensitivity associated with the alternative VOI inspection scenarios.

## 4. Discussion

A change from CMI to VOI has the potential to substantially impact on bTB control and eradication programmes in non-OTF countries. The four VOI inspection scenarios, with progressive reduction in animal-level inspection sensitivity, lead to substantial changes in the effectiveness of abattoir surveillance, as measured by herd-level detection sensitivity (Figures 1–5, Table 4)**.** It is clear from the graphs, that the distributions gradually become more and more skewed towards the lower herd-sensitivities as animal-level inspection sensitivity decreases, since the many herds shipping only one animal during the 3 year study period have a certain low probability of being detected as bTB-infected. Taking VOI3 as an example (with a 3-fold reduction in animal-level inspection sensitivity compared to CMI), the herd-level detection sensitivity compared to CMI would drop by 50%, if mean sensitivity estimates are compared, and by 64%, if median sensitivity estimates are compared (Table 4).

The estimated herd-level detection sensitivity of CMI, 0.24, is low and a substantial number of bTB-herds are not currently being detected during abattoir surveillance (Table 4). Herd-level detection sensitivity is particularly influenced by the large number of herds that send on only small numbers of animals to the factory (mode = 1) (Table 2), and the very low true within-herd prevalence in infected herds (median = 0.02). Further, herd-level detection sensitivitye is reduced under each of the simulated scenarios, from 0.16 for VOI2 to 0.08 for VOI5 (Table 4), which increases the estimated number of bTB-herds that would not be detected. With a change from CMI to VOI3, an estimated additional 1,880 bTB-herds (depending on use of estimates based on either mean or median values), being 48% of those detected under CMI, would remain non-detected during abattoir surveillance in Ireland during 2010–2012 (Table 4). The absolute number of infected herds needs to be interpreted with care, as discussed later, however the trends are clear. Abattoir surveillance plays a critical role in national eradication programmes in non-OTF countries, particularly in Ireland where cattle movement is frequent (45): through early detection of infected herds, and through detection of animals with lesions but non-responsive to the skin test (9). We accept, however, that the impact of this non-detection on the national eradication programme will be minimal in many cases as a result of infected cattle herds identified subsequently during annual field surveillance, as all cattle greater than 6 weeks of age are tested annually using the SICTT (12). However, there will be an impact on programme progress if infection is transmitted to other animals prior to annual field surveillance, either in the same herd or following movement to other herds.

The study results need to be considered in the context of a series of methodological challenges. The estimated number of bTB-herds (that is, those infected) as presented in Table 4 is substantially greater than previously reported (16) and likely too high to be realistic, with several possible explanations. For the purpose of this study, true within-herd prevalence for infected herds was estimated from the apparent prevalence of the number of FLTs for each herd divided by the number of animals sent to slaughter. However, this is likely to have underestimated prevalence due to the inherent sampling bias that is associated with abattoir surveillance. bTB risk is known to increase with age (46), however animals presented to slaughter in Ireland include a large number of young animals from beef herds (representing an estimated 80% of herds in Ireland) and old, cull cows from dairy. Further, we have assumed that the sensitivities of meat inspection and of bacteriology are statistically independent, whereas this may not be the case given that a positive bacteriological result is more likely in animals with gross pathology compared to those without. Although many herds in Ireland are small, in this study we have used a binomial (rather than a hypergeometric) distribution to reflect sampling of animals from the study herds because the number of animals slaughtered during the study period is generally very low (with a mode of 1; Table 2). In this study, we focus solely on abattoir surveillance in non-restricted herds, as it is in these herds in non-OTF countries where abattoir surveillance is particularly important. For this reason, our definitions of bTB-herd, apparent herd prevalence (AP_h_) and apparent within-herd prevalence (AP_wh_) (see Table 1) each refer to Irish herds subject to routine abattoir surveillance during 2010–12, that is, whilst unrestricted and free to trade. We have assumed that each FLT was triggered by a single lesioned animal, however, it is possible, but unlikely, that more than one lesioned animal from one and the same herd was detected at the same time. During the study period, 4,043 (4.7%) eligible herds experienced at least one FLT, including 7 herds with 4 FLTs (Table 3). Among non-restricted herds presenting with a confirmed bTB lesion at slaughter in Ireland, there is no evidence of within-herd transmission in about 80% of these herds (14). In a recent study, previous bTB exposure was identified as a key risk factor for animals presenting with confirmed bTB lesions as slaughter but without any evidence of within-herd transmission (47). In the absence of any reactors at the FLT, these herds will be restricted once one further clear whole-herd test is conducted. Given this background, it is entirely plausible that a small number of herds could have had 4 FLTs during the three year study period. Finally, this work is based on estimates from the literature concerning the sensitivities of meat inspection and bacteriology. With respect to animal-level inspection sensitivity, this is likely to vary greatly, both between and within non-OTF countries. In the current study, we have estimated a median animal-level inspection sensitivity (that is, the probability that bTB-like lesions will be observed in a bTB-infected animal during abattoir surveillance) of 55%, based on available published work. Previously, Foddai (11) used a pert distribution of (90%, 95%, 99%) to represent the probability under CMI that a veterinarian will detect lesions when these are present. In earlier work from Ireland (48, 49) and the UK (13), substantial abattoir-level differences in submission and confirmation rates have been identified. Factors likely to affect animal-level inspection sensitivity include physical factors, such as line speed and light intensity, and human factors, such as the quality of inspection, as influenced by the competence of the inspector (50).

There were differences between study herds with at least one FLT and all study herds with respect to the number of cattle sent to slaughter (Table 2). This could be a reflection of the probability of bTB occurrence, which is known to be higher in larger herds than in smaller herds. Many studies have identified herd size as a risk factor for bTB. The reasons for this are not entirely understood. As noted previously (15), increasing herd size may increase opportunity for exposure, both within the herd and from neighboring herds (51, 52). However, herd-level detection sensitivity is heavily driven by the number of animals sampled, therefore a greater probability of detection is to be expected in herds supplying greater numbers of animals to slaughter, even if the underlying probability of herd-level infection were independent of the number of animals slaughtered. As can be seen from the formula for herd sensitivity and from the simulations in Table 4 and Figure 6, herd-level detection sensitivities are invariably lower than animal-level detection sensitivities. Herd-level sensitivities are not only dependent on the animal-level sensitivities, since other factors are also important (18), in particular, the number of animals tested (in this case, animals sent for slaughter) from the herds in question. Of course, also the higher the within-herd prevalence, the higher the herd-level detection sensitivity, although the variability of this parameter is beyond the analyses considered in the present study.

In the EFSA opinion (8), animal-level inspection sensitivity was primarily considered when seeking to identify the effects of potential changes in meat inspection procedures. In the current paper, we have extended these earlier concepts to distinguish the animal-level sensitivity of meat inspection alone and in series with bacteriology, noting that the latter detection methodology (or alternatively meat inspection and histopathology) is routinely used in Ireland (12) for bTB-detection at slaughter. Further, from a programme perspective, knowledge of herd-level detection sensitivity is important, providing an estimate of the probability that *M. bovis* will be detected in one or more animals from an infected but non-restricted herd during abattoir surveillance. Each of these terms is defined in the glossary (Table 1).

This work highlights the importance of abattoir surveillance in non-OTF countries, to facilitate both the early detection of infected herds and the detection of infection in herds with one or more animals with bTB lesions but without animals positive to the skin test. In OTF countries, abattoir surveillance is also extremely important, forming the basis of national surveillance to substantiate bTB free status (11). In each case, animal-level inspection sensitivity greatly influences the desired surveillance outcome. We speculate, however, that reasonable levels of animal-level inspection sensitivity may be harder to achieve in OTF countries, where inspectors rarely encounter, and are thus unfamiliar with, bTB or bTB-like lesions. In such situations, a number of strategies have been introduced to maximise the sensitivity of meat inspection. In Australia, which is bTB-free, efforts include raising awareness, encouraging submissions from, and providing feedback to, meat inspectors, and using risk-based sampling and submission targets (53). In the US, where bTB is rare, an incentive programme is used to increase the number of tissues during abattoir surveillance that are submitted for laboratory examination (9).

There have been recent changes to EU legislation relevant to meat inspection of pigs, including the visual inspection of pig carcasses and offal by officials at post-mortem (54), in association with strengthened process hygiene criteria for *Salmonella* (55) and a more risk-based *Trichinella* testing regime (56). In contrast, to this point meat inspection of cattle carcasses continues to be conducted using CMI. There are likely differences between species with respect to the microbial hazard associated with CMI, and the public health gains that would accrue with a shift to VOI. To the authors’ knowledge, however, these differences have not yet been quantified.

If VOI were introduced in EU cattle abattoirs without alternative surveillance means to compensate for the decrease in the herd-level detection sensitivity of abattoir surveillance, such changes might jeopardise bTB surveillance, control and eradication programmes in non-OTF countries, including Ireland. Based on the results of a recent study (11), the impact of such changes in OTF countries would be less. In Denmark, an OTF country, confidence of freedom could be maintained even if VOI were to replace CMI, but only if the annual probability of bTB introduction were kept very low.

## Author Contributions

PW designed the study and monitored the compilation of the datasets and the estimates from these to be used as basis for the simulations. CM developed the simulation program based on the datasets and prepared and completed the simulation procedures. EH contributed to the analytical design, presentation and interpretation of data for the simulations. IH and TC carried out the preparation, analysis and presentation of the slaughter data and results for further analysis. SM provided access to and interpretation of the Irish slaughterhouse data, and provided literature as the basis for the sensitivity estimates of CMI. PW and SM drafted the initial manuscript, and all co-authors reviewed the work critically, suggested revisions and finally approved the version to be published.

## Conflict of Interest Statement

The authors declare that the research was conducted in the absence of any commercial or financial relationships that could be construed as a potential conflict of interest

## Appendix 1: R code to estimate herd-level detection sensitivity (HSe)

set.seed(1)

 n <- sample(× = data$Sum_Slaughtered2010_2012, size = 5000, replace = F) 

herd.df <- data.frame(nTested = numeric(), Herd.inf = numeric(), Herd.det = numeric()) 

sims = 1000

###Animal level Se (ASe)

Se = 0.42 ###baseline

#Se = 0.462 ###10%

#Se = 0.378 ###−10%

###ASe multiplication factor

Red = 1.0

#Red = 0.5

#Red = 0.333

#Red = 0.25

#Red = 0.2

for(j in n){ 

df <- data.frame(nSlt = numeric(), inf = numeric(), detect = numeric())

for(i in 1:sims){

#inf <- rbinom(1, 1, runif(1, 0.0368, 0.0471)) ### HTP (only relevant for Sp <1)

inf <- 1

tp <- rbeta(1, 1.259818, 37.929349) ###fitted from within-herd TP distribution 

ap <- inf*Se*tp*Red

npos <- rbinom(*n*=1, size=j, prob=ap)

detect <- ifelse(npos >0, 1, 0)

A <- data.frame(inf=inf, nSlt=j, detect=detect)

df <- bind_rows(df, A)

}

B <- data.frame(nTested=j, Herd.inf=sum(df$inf), Herd.det=sum(df$detect)) herd.df <- bind_rows(herd.df, B)

}

herd.df$Se <- herd.df$Herd.det/herd.df$Herd.inf
